# The Central Funds and Special Hospitals

**Published:** 1904-12-03

**Authors:** 


### THE CENTRAL FUNDS AND SPECIAL HOSPITALS.

WE note that our contemporary, *The Medical Press*, in a long leading article in its issue of the 30th ult., avoids the points raised by its former article referred to in our issue of the 19th ult. In that article it will be remembered that *The Medical Press* asserted in effect that the policy of the Sunday and King Edward's Hospital Funds "has resulted in the withholding of grants and injury to the reputation of hosts of small but deserving medical charities." We invited our contemporary to produce a list and particulars of these "hosts of small but deserving medical charities." After 10 days' consideration all it has to say on this point is that "it hopes to furnish the desired list of small hospitals in an early issue." This is a very unsatisfactory way of dealing with a great question. Either the writer of the original article knew what he was writing about, and had full particulars before him on which his article was based, or he did not know the facts, and is not even able, after 10 days' interval, to produce them. Unless his "hosts of small hospitals" have been working underground and unknown to the people of London, we make bold to anticipate that it will be found that they have little or no existence in fact. We have some confidence that the editor of our contemporary will not allow any writer to use the columns of *The*

*Medical Press* to champion the private promoter who for his own purposes may hire indifferent premises and paint the word "Hospital" on their face. If this be so, then no doubt it will be found that the list which the writer of the original article, after further inquiry, may evolve, may not contain the name of a single reputable hospital which will bear the test of inspection, or which can, in any sense, be described as a modern hospital, efficient and up to date in all departments.

At any rate we are entitled to insist that if the writer responsible for the original statement does not produce full particulars of "the hosts of small but deserving medical charities" which are trembling to their fall, owing to the action of the Sunday and King Edward's Hospital Funds, the original charge must be unreservedly withdrawn.

The second article bears on its face the source from which it springs, from the circumstance that it relates almost entirely to the case of the Royal Orthopædic Hospital and the dispute which arose as to the sale of its site in Oxford Street. That sale was opposed by a small minority only of the Governors, as the voting showed, unless we are greatly mistaken, and no one who inspected the hospital in Oxford Street, and realised how the work was carried on there, could doubt for one moment that the majority of the Governors were right in the decision to which they came. The writer falls foul of the Sunday Fund authorities for having urged the sale of this site, and claims that had they had their way the Royal Orthopædic Hospital must have lost £12,000. This interesting statement seems to be based on the fact that the ultimate price obtained for the site was £12,000 more than that which was contained in an offer which was under the consideration of the Governors at the time the Hospital Sunday Fund urged that a sale was desirable. How any unprejudiced writer in a medical journal can attack the Hospital Sunday or any other public authority on such grounds passes understanding. It was the duty of the Governors to so conduct their business as to secure the highest obtainable price for the site. The Sunday Fund could have had nothing to do with the price or the value of the site, and their action must have been confined to an expression of opinion in favour of its being realised and a new site found elsewhere.

We have no special knowledge of the proceedings of the Sunday Fund, but on the statement made by *The Medical Press* it is perfectly clear that the Sunday Fund Council did not, as on public grounds they could not, take any direct part in the sale, or have anything whatever to do with the price obtained. Our contemporary expresses a desire for information as to the principles that prompt the committees of the two funds in making their awards, and this information can no doubt be obtained on application to the proper authorities. It has little bearing upon the original charge of *The Medical Press*, however, and that charge, as we have said, must be supported by evidence, or unreservedly withdrawn. That is our contention in the interests of fair comment, the public, and the supporters of hospitals generally. We are confident the editor of *The Medical Press* will make it his business, for the reputation of his paper, to see that one of these courses is taken in its next issue.

